# Diagnostic performance of salivary gland ultrasonography using the OMERACT scoring system in Sjögren disease: A cross-sectional diagnostic accuracy study

**DOI:** 10.1177/1759720X261480003

**Published:** 2026-08-27

**Authors:** Md. Ismail Hossain, Minhaj Rahim Choudhury, Muhammad Shoaib Momen Majumder, Md. Nahiduzzamane Shazzad, Mohammad Abul Kalam Azad, Md. Masudul Hassan, Rijwan Bhuiyan, Md. Shohel Hasan

**Affiliations:** 1 Department of Rheumatology, 469135 Rajshahi Medical College, Rajshahi, Bangladesh; 2 Department of Rheumatology, 74464 Bangladesh Medical University, Dhaka, Bangladesh; 3 Department of Public Health and Informatics, 74464 Bangladesh Medical University, Dhaka, Bangladesh

**Keywords:** Sjögren disease, salivary gland ultrasonography, OMERACT, diagnostic accuracy, Schirmer’s test, unstimulated whole salivary flow rate

## Abstract

**Background:**

Sjögren disease (SjD) is an autoimmune disorder characterized by salivary and lacrimal gland dysfunction. Salivary gland ultrasonography (SGUS) is a non-invasive, accessible imaging modality for assessing glandular structural changes, although its diagnostic performance compared with conventional diagnostic tests requires further evaluation.

**Objectives:**

To evaluate the diagnostic accuracy of OMERACT-scored salivary gland ultrasonography in patients with suspected primary or secondary SjD.

**Design:**

This is a cross-sectional diagnostic accuracy study of salivary ultrasonography.

**Methods:**

This study was conducted in the Department of Rheumatology, Bangladesh Medical University, from February 2023 to February 2024. Patients presenting with sicca symptoms were classified according to the 2016 ACR/EULAR classification criteria, which served as the reference standard. All participants underwent clinical evaluation, Schirmer’s test, unstimulated whole salivary flow rate (UWSFR), anti-SSA/anti-SSB antibody testing, and SGUS of the parotid and submandibular glands. SGUS images were scored using the OMERACT semiquantitative grayscale scoring system (0–3 per gland) by a blinded examiner. Diagnostic performance was assessed using sensitivity, specificity, and receiver operating characteristic (ROC) curve analysis.

**Results:**

Among 100 participants, 50 fulfilled the 2016 ACR/EULAR classification criteria for SjD. Patients with SjD had significantly higher mean SGUS scores and lower mean UWSFR compared with those without SjD (*p* < 0.001). An OMERACT SGUS grade ≥2 in at least one gland demonstrated 84% sensitivity and 92% specificity. A total SGUS score ≥4 showed 88% sensitivity and 86% specificity, whereas a score ≥6 provided 100% specificity with reduced sensitivity (64%). SGUS showed excellent diagnostic performance with an area under the ROC curve (AUC) of 0.94 (95% CI: 0.90–0.99). Compared with SGUS, Schirmer’s test demonstrated lower diagnostic accuracy (sensitivity 65.3%, specificity 52%), while UWSFR showed high sensitivity (92%) but limited specificity (48%). SGUS score showed a weak positive correlation with disease duration (*r* = 0.33, *p* < 0.05).

**Conclusion:**

OMERACT-scored salivary gland ultrasonography demonstrated excellent diagnostic accuracy and superior specificity compared with conventional functional tests. SGUS may serve as a valuable non-invasive adjunctive tool in the diagnostic evaluation of patients with suspected SjD.

## Plain language summary

Sjögren disease is an autoimmune condition that commonly causes dry eyes and dry mouth and can be difficult to diagnose. This study evaluated whether salivary gland ultrasonography (SGUS), a simple, noninvasive imaging technique, can help identify the disease.

We assessed 100 patients with symptoms suggestive of Sjögren disease using standard diagnostic tests and ultrasound examination of the major salivary glands. Ultrasound findings were scored using the OMERACT grading system and compared with the 2016 ACR/EULAR classification criteria.

Patients with Sjögren disease had significantly higher ultrasound scores than those without the disease. SGUS showed high diagnostic accuracy and outperformed conventional functional tests in distinguishing affected from unaffected individuals.

These findings suggest that OMERACT-scored SGUS is a reliable, non-invasive, and readily available tool that may improve the diagnostic evaluation of patients with suspected Sjögren disease.

## Introduction

Sjögren disease (SjD) is a chronic systemic autoimmune disease characterized by lymphocytic infiltration of the exocrine glands, resulting in sicca symptoms, including dry eyes and dry mouth. The disease may occur as a primary condition or in association with other autoimmune diseases (secondary SjD). Beyond exocrine gland dysfunction, SjD may involve multiple organ systems and is associated with an increased risk of B-cell non-Hodgkin lymphoma.^1,2^

The 2016 American College of Rheumatology/European League Against Rheumatism (ACR/EULAR) classification criteria provide a standardized framework for classifying SjD and demonstrate high diagnostic accuracy.^3^ Nevertheless, important limitations remain in routine clinical practice. Minor salivary gland biopsy (MSGB), although a key component of the classification criteria, is invasive, subject to sampling variability, and may not be feasible in all patients.^4,5^ Likewise, serological markers, particularly anti-SSA antibodies, and conventional objective tests, including Schirmer’s test and unstimulated whole salivary flow rate (UWSFR), demonstrate variable diagnostic performance when used individually and should be interpreted within the overall clinical context.^2,3^

UWSFR is widely used to evaluate salivary gland function, with hyposalivation defined as ≤0.100 mL/min. However, functional impairment does not necessarily reflect the extent of structural damage to the salivary glands, highlighting the need for complementary imaging modalities that can directly assess glandular morphology.^6^

Salivary gland ultrasonography (SGUS) has emerged as a non-invasive, readily available, reproducible, and cost-effective imaging technique for evaluating structural abnormalities of the major salivary glands in patients with suspected Sjögren disease.^7^ The Outcome Measures in Rheumatology (OMERACT) semiquantitative scoring system has improved the standardization, reliability, and reproducibility of SGUS assessment, facilitating its implementation in both clinical practice and research.^8^

Recent systematic reviews and clinical studies have demonstrated that SGUS has good diagnostic accuracy for SjD. However, its incremental diagnostic value relative to conventional serological and functional assessments remains incompletely established, particularly in routine clinical practice and across diverse patient populations.^7,9^ Consequently, further validation is required before SGUS can be routinely incorporated into diagnostic algorithms.

Accordingly, this study aimed to evaluate the diagnostic accuracy of OMERACT-scored salivary gland ultrasonography in patients with suspected SjD. In addition, the diagnostic performance of SGUS was compared with that of Schirmer’s test, unstimulated whole-salivary flow rate, and anti-SSA antibodies, using the 2016 ACR/EULAR classification criteria as the reference standard.

## Methods

### Study design and setting

**Figure 1.**
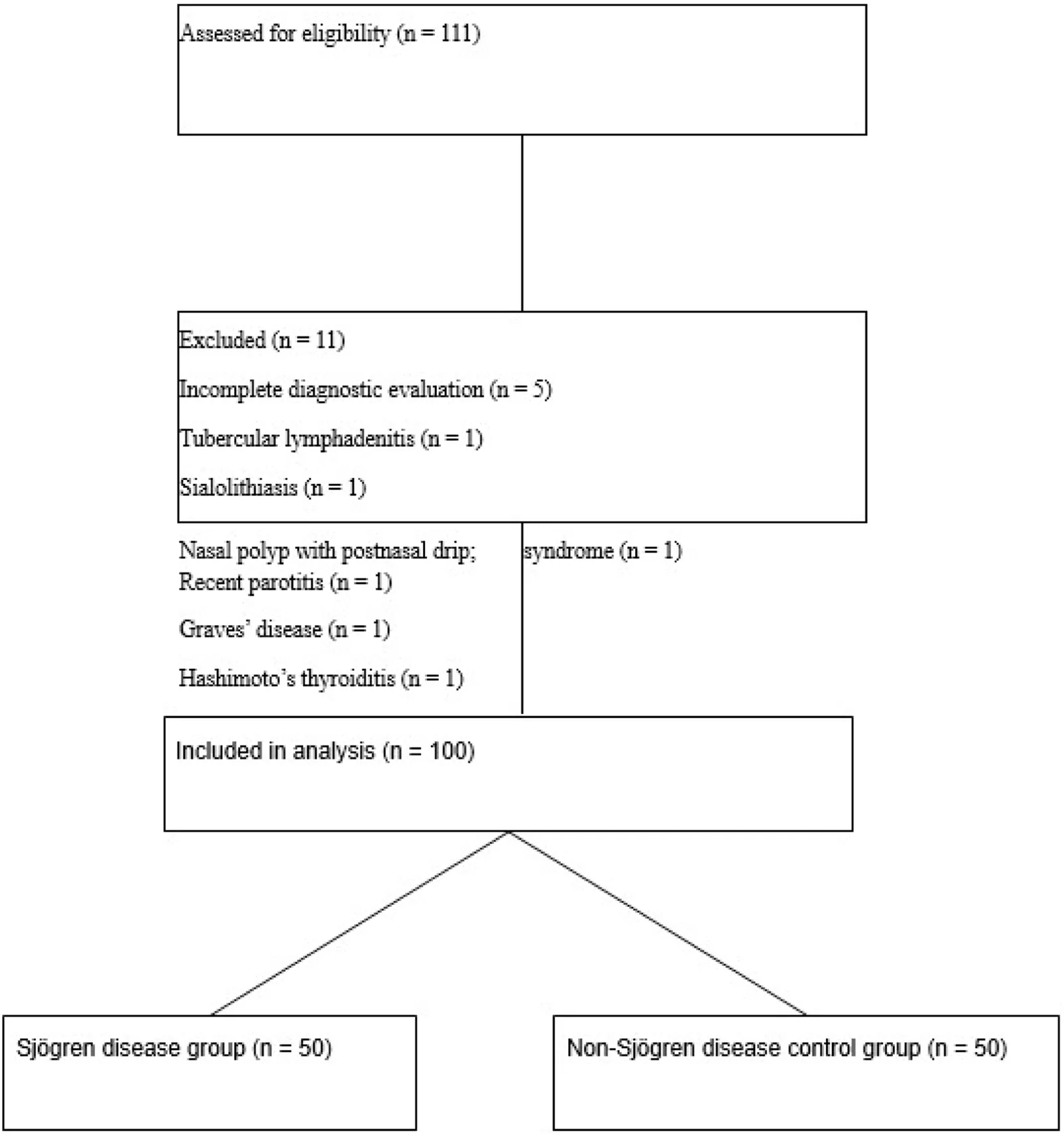
Flow of participants in the study (STROBE flow diagram). A total of 111 participants were assessed for eligibility. Eleven patients were excluded (five due to incomplete diagnostic evaluation and six due to alternative diagnoses). The remaining 100 participants were included in the analysis and subsequently classified into the Sjögren disease group (*n* = 50) and the non-Sjögren disease control group (*n* = 50).

This cross-sectional diagnostic accuracy study was conducted in the Department of Rheumatology at Bangladesh Medical University (BMU), Dhaka, Bangladesh, from February 2023 to February 2024. The study was reported in accordance with the Standards for Reporting of Diagnostic Accuracy Studies (STARD) guidelines and the Strengthening the Reporting of Observational Studies in Epidemiology (STROBE) statement.^10,11^ A participant flow diagram is presented in Figure 1.

*The study protocol was reviewed and approved by the Institutional Review Board (IRB) of Bangladesh Medical University (formerly Bangabandhu Sheikh Mujib Medical University), Dhaka, Bangladesh (Approval No. BSMMU/2023/10935). Written informed consent was obtained from all participants before enrollment. The study was conducted in accordance with the ethical principles of the Declaration of Helsinki*.

### Study population and recruitment

Consecutive adult patients presenting with ocular and/or oral sicca symptoms were recruited during the study period to minimize selection bias. All eligible participants underwent standardized clinical, laboratory, and imaging assessments during a single study visit.

Participants were classified into the SjD and non-SjD groups according to the 2016 American College of Rheumatology/European League Against Rheumatism (ACR/EULAR) classification criteria, which served as the reference standard.^3^ Patients fulfilling the 2016 ACR/EULAR classification criteria were classified as having either primary or secondary SjD. In contrast, those who did not meet the criteria were classified as non-SjD controls.

Diagnostic delay was defined as the interval between the patient-reported onset of sicca symptoms and the date of diagnosis of SjD.

#### Inclusion criteria

Participants were eligible if they were aged ≥18 years, presented with ocular and/or oral sicca symptoms suggestive of SjD, and provided written informed consent.

#### Exclusion criteria

Patients were excluded if they had incomplete clinical, serological, or imaging data; declined participation; or had conditions unrelated to SjD that could substantially alter salivary gland structure or interfere with ultrasonographic assessment.

#### Index test

The index test was SGUS performed using the OMERACT semiquantitative grayscale scoring system.

#### SGUS acquisition

Ultrasonography of the bilateral parotid and submandibular glands was performed using a high-frequency linear transducer (Mindray DC-80 X-Insight; L14-6 probe). Participants were examined in the supine position with slight neck extension and gentle head rotation. Longitudinal and transverse scans of each gland were obtained in accordance with standardized scanning protocols.^12^ To avoid incorporation bias, SGUS findings were not considered when applying the 2016 ACR/EULAR classification criteria and were not used to establish the reference-standard diagnosis.

#### Image interpretation

All SGUS examinations were independently interpreted by two experienced rheumatologists who were EULAR-certified musculoskeletal sonologists and APLAR trainers in musculoskeletal ultrasonography. Both readers were blinded to the participants’ clinical findings, laboratory results, and final diagnosis. Ultrasonographic images were digitally stored and reviewed independently. Any discrepancies in image interpretation or scoring were resolved by consensus to establish the final SGUS score.

#### Scoring system

SGUS findings were graded using the OMERACT semiquantitative grayscale scoring system, with each parotid and submandibular gland assigned a score ranging from 0 to 3.^8^ Grade 0 indicated normal parenchyma; grade 1, mild parenchymal inhomogeneity without focal anechoic or hypoechoic areas; grade 2, moderate inhomogeneity with focal anechoic or hypoechoic areas; and grade 3, severe diffuse inhomogeneity with extensive anechoic or hypoechoic areas and/or fibrotic changes. Grades 0–1 were considered normal or non-specific, whereas grades 2–3 were considered pathological and suggestive of SjD. Total SGUS scores were calculated by summing the scores of the examined glands.

#### Reference standard

The reference standard was the diagnosis of SjD according to the 2016 ACR/EULAR classification criteria.^3^ These criteria incorporate weighted objective measures, including anti-SSA antibody positivity, minor salivary gland histopathology, ocular staining score, Schirmer’s test, and UWSFR.

#### Minor salivary gland biopsy

MSGB was performed only in selected patients with negative anti-SSA antibodies and/or persistent diagnostic uncertainty despite comprehensive clinical assessment, serological evaluation, and objective functional testing. Owing to its invasive nature, biopsy was not performed routinely but was reserved for cases in which the findings were expected to influence the final diagnosis. A 4-mm punch biopsy of the lower lip was performed under local anesthesia, and an experienced pathologist undertook histopathological examination. A focus score of ≥1 focus per 4 mm^2^ of glandular tissue was considered diagnostic for SjD.^4,5,13^

### Additional diagnostic assessments

All participants underwent standardized assessments in accordance with the study protocol. Schirmer’s test was performed without topical anesthesia, and a wetting of ≤5 mm after 5 minutes was considered positive, consistent with the 2016 ACR/EULAR classification criteria.^3^ UWSFR was measured using the standardized spitting method over 15 minutes, with hyposalivation defined as a flow rate of ≤0.100 mL/min.^6^

Serological investigations included antinuclear antibody (ANA) testing by HEp-2 indirect immunofluorescence, extractable nuclear antigen (ENA) profile by line immunoassay, anti-SSA/Ro and anti-SSB/La antibodies by chemiluminescent immunoassay (CLIA), rheumatoid factor (RF), anti-cyclic citrullinated peptide (anti-CCP) antibodies, and anti-double-stranded DNA (anti-dsDNA) antibodies. These investigations served as comparator diagnostic modalities.

### Study procedures and blinding

All clinical, laboratory, and ultrasonographic assessments were completed during a single study visit. Rheumatologists performing and interpreting SGUS were blinded to the participants’ clinical findings and laboratory results. Laboratory personnel were blinded to the ultrasonographic findings. The 2016 ACR/EULAR classification criteria were applied independently of the SGUS results to minimize incorporation bias.

### Outcomes

The primary outcome was the diagnostic accuracy of OMERACT-scored salivary gland ultrasonography for identifying SjD, expressed as sensitivity, specificity, positive predictive value (PPV), negative predictive value (NPV), and their corresponding 95% confidence intervals (CIs).

Secondary outcomes included determination of the optimal SGUS cutoff value using receiver operating characteristic (ROC) curve analysis, estimation of the area under the ROC curve (AUC), comparison of the diagnostic performance of SGUS with Schirmer’s test, UWSFR, and anti-SSA antibody positivity, and evaluation of the association between SGUS scores and disease duration.^14,15^

#### Sample size estimation

The sample size was calculated using the Buderer formula for diagnostic accuracy studies, which incorporates the anticipated sensitivity, disease prevalence, desired precision, and confidence level.^16^ Assuming an anticipated sensitivity of 82% for salivary gland ultrasonography based on previous studies,^17^ a disease prevalence of 50% among clinically suspected patients, a 95% CI, and an absolute precision of 10%, the minimum required sample size was estimated to be 114 participants. However, owing to the limited number of eligible patients during the study period, 100 consecutive participants meeting the inclusion criteria were enrolled. Although this sample was slightly smaller than the calculated requirement, it was considered adequate to estimate the overall diagnostic performance of salivary gland ultrasonography with acceptable precision. Nevertheless, the smaller sample size may have resulted in wider confidence intervals, and the study was not powered for subgroup analyses.

#### Statistical analysis

Statistical analyses were performed using IBM SPSS Statistics for Windows, version 25.0 (IBM Corp., Armonk, NY, USA). Continuous variables were assessed for normality and are presented as mean ± standard deviation (SD) or median with interquartile range (IQR), as appropriate. Categorical variables are presented as frequencies and percentages.

Comparisons between the SjD and non-SjD groups were performed using Student’s *t*-test for normally distributed continuous variables or the Mann–Whitney *U* test for non-normally distributed variables. Categorical variables were compared using the chi-square test or Fisher’s exact test, as appropriate.

The diagnostic performance of SGUS and comparator tests was evaluated by calculating sensitivity, specificity, PPV, NPV, positive likelihood ratio (LR+), negative likelihood ratio (LR−), overall diagnostic accuracy, and their corresponding 95% confidence intervals (CIs). ROC curve analysis was performed to assess the diagnostic performance of SGUS, determine the optimal cutoff values, and calculate the area under the ROC curve (AUC) with 95% CI. All statistical tests were two-tailed, and a *p*-value <0.05 was considered statistically significant.

## Results

### Baseline characteristics

A total of 111 patients were screened. After excluding 11 patients (incomplete evaluation, *n* = 5; alternative diagnoses, *n* = 6), 100 participants were included in the final analysis: 50 with SjD and 50 without SjD (Figure 1).

In the SjD group, 58% of patients reported spontaneous sicca symptoms, whereas in 42% the symptoms were identified only after directed clinical questioning. The mean diagnostic delay, defined as the interval from symptom onset to diagnosis, was 6.2 ± 6.3 years.

Dental loss (88%), parotid enlargement (38%), and reduced salivary pooling (80%) were significantly more frequent in SjD patients compared with non-SjD participants (*p* < 0.001) (Table 1).

**Table 1.** Baseline characteristics of the study participants (*n* = 100).

Characteristic	Overall (*N* = 100)	SjD (*n* = 50)	Non-SjD (*n* = 50)	*P* value
**Age, years** *	42.5 ± 12.0	39.1 ± 12.4	46.0 ± 10.7	0.004^a^
**Age at symptom onset, years** *	37.5 ± 11.9	33.5 ± 11.7	42.3 ± 10.3	<0.001^a^
**Age at diagnosis, years** *	42.0 ± 12.3	39.2 ± 12.3	45.8 ± 11.4	0.012^a^
**Sex**	​	​	​	0.999^b^
Male	3 (3.0)	1 (2.0)	2 (4.0)	​
Female	97 (97.0)	49 (98.0)	48 (96.0)	​
**Sicca symptom reporting**	​	​	​	0.840^c^
Voluntary	59 (59.0)	29 (58.0)	30 (60.0)	​
On directed questioning	41 (41.0)	21 (42.0)	20 (40.0)	​
**Duration of dry mouth, years** *	2.0 ± 2.7	2.7 ± 3.4	1.3 ± 1.6	0.008^a^
**Duration of dry eye symptoms, years** *	1.9 ± 3.4	2.8 ± 4.4	0.9 ± 1.4	0.013^a^
**Disease duration, years** *	5.2 ± 5.5	6.2 ± 6.3	4.1 ± 4.2	0.085^a^
Dry lips/shiny buccal mucosa present	84 (84.0)	48 (96.0)	36 (72.0)	0.002^b^
Dental loss present	72 (72.0)	44 (88.0)	28 (56.0)	0.001^c^
Parotid enlargement present	21 (21.0)	19 (38.0)	2 (4.0)	0.001^b^
Sublingual salivary pooling present	41 (41.0)	10 (20.0)	31 (62.0)	0.001^c^

SjD indicates Sjögren’s disease.

^*^Mean (± standard deviation).

^a^Independent *t* test.

^b^Fisher’s Exact test.

^c^Chi-square test.

#### Laboratory and imaging findings

The total SGUS score was significantly higher in the SjD group compared with the non-SjD group (6.6 ± 2.7 vs. 1.6 ± 1.4; *P* < 0.001). UWSFR was significantly lower in SjD patients (0.072 ± 0.090 vs. 0.116 ± 0.108 mL/min; *P* < 0.001).

No significant difference was observed in Schirmer’s test positivity between groups (65.3% vs. 48.0%; *P* = 0.082). Anti-SSA antibody positivity was significantly higher in the SjD group (87.8% vs. 0%; *P* < 0.001) (Table 2).

**Table 2.** Diagnostic parameters of the study participants (*n* = 100).

Parameter	Overall (*N* = 100)	SjD (*n* = 50)	Non-SjD (*n* = 50)	*P* value
Schirmer’s test positive	56 (56.0)	32 (65.3)	24 (48.0)	0.082^a^
UWSFR, mL/min*	0.094 ± 0.101	0.072 ± 0.090	0.116 ± 0.108	<0.001^b^
Total SGUS score*	4.12 ± 3.30	6.6 ± 2.7	1.6 ± 1.4	<0.001^b^
Anti-SSA (Ro) positive	43 (43.0)	43 (87.8)	0 (0.0)	<0.001^c^
Anti-SSB (La) positive	8 (8.0)	8 (16.3)	0 (0.0)	0.003^c^
Antinuclear antibody (ANA)	​	​	​	<0.001^c^
Weakly positive	1 (1.0)	1 (2.0)	0 (0.0)	​
Moderately positive	27 (27.0)	15 (30.6)	12 (24.5)	​
Strongly positive	29 (29.0)	27 (55.1)	2 (4.1)	​
Negative	41 (41.0)	6 (12.2)	35 (71.4)	​
Rheumatoid factor positive	24 (24.0)	12 (24.0)	12 (24.0)	0.990^a^
ACPA positive	14 (14.0)	7 (14.0)	7 (14.0)	0.990^a^
Minor salivary gland biopsy	​	​	​	0.117^c^†
Not performed	91 (91.0)	44 (88.0)	47 (94.0)	​
Focus score ≥1	4 (4.0)	4 (8.0)	0 (0.0)	​
Focus score <1	5 (5.0)	2 (4.0)	3 (6.0)	​

All are numbers (%) unless otherwise indicated.

*Mean (±standard deviation).

^a^Independent t-test for comparison of continuous variables between groups.

^b^Chi-square test for comparison of categorical variables.

^c^Fisher’s exact test for categorical variables when expected cell counts were <5. UWSFR, unstimulated whole salivary flow rate; SGUS, salivary gland ultrasonography; ANA, antinuclear antibody; ACPA, anti-citrullinated protein antibody; SD, standard deviation.

MSGB was performed in 9 anti-SSA–negative patients with high clinical suspicion or diagnostic uncertainty. Among them, 4 patients had a positive biopsy (focus score ≥1). Two biopsy-negative patients nevertheless fulfilled the 2016 ACR/EULAR classification criteria based on anti-SSA positivity and other objective clinical findings.

#### Diagnostic performance of SGUS

An OMERACT SGUS grade ≥2 in at least one gland demonstrated a sensitivity of 84.0%, specificity of 92.0%, PPV of 91.3%, and NPV of 85.2%.

A total SGUS score ≥4 yielded a sensitivity of 88.0% and specificity of 86.0%, whereas increasing the cutoff to ≥6 improved specificity to 100% but reduced sensitivity to 64.0%.

Overall, the total SGUS score demonstrated excellent diagnostic accuracy for SjD, with an area under the ROC curve (AUC) of 0.94 (95% CI: 0.90–0.99) (Table 3).

**Table 3.** Diagnostic performance of salivary gland ultrasonography (SGUS) cut-offs using the 2016 ACR/EULAR criteria as the reference standard (*n* = 100).

SGUS cut-off	Sensitivity	Specificity	PPV	NPV
	% (95% CI)	% (95% CI)	% (95% CI)	% (95% CI)
SGUS ≥2 in ≥1 gland	84.0 (70.9–92.8)	92.0 (80.8–97.8)	91.3 (80.3–96.4)	85.2 (75.2–91.6)
Total SGUS score ≥4	88.0 (75.7–95.5)	86.0 (73.3–94.2)	86.3 (75.8–92.6)	87.8 (77.0–93.9)
Total SGUS score ≥6	64.0 (49.2–77.1)	100.0 (92.9–100.0)	100.0 (89.1–100.0)	73.5 (65.8–80.0)

CI, confidence interval; PPV, positive predictive value; NPV, negative predictive value; Diagnostic performance was calculated using the 2016 ACR/EULAR classification criteria as the reference standard; Confidence intervals were calculated using the exact (Clopper–Pearson) method; The limited sample size may influence the high specificity observed at higher SGUS cut-off values.

ROC analysis of individual salivary glands showed consistently excellent diagnostic performance, with AUC values ranging from 0.93 to 0.95.

#### Comparison with conventional diagnostic tests

Anti-SSA antibodies showed a sensitivity of 87.8% and specificity of 100%, demonstrating high diagnostic specificity for SjD.

UWSFR demonstrated the highest sensitivity (92.0%) but low specificity (48.0%), whereas Schirmer’s test showed lower sensitivity (65.3%) and specificity (52.0%).

Overall, SGUS demonstrated higher specificity and diagnostic accuracy than functional tests and showed diagnostic performance comparable to that of anti-SSA serology (Table 4).

**Table 4.** Diagnostic performance of SGUS, UWSFR, Schirmer’s test, and anti–SS-A antibodies using 2016 ACR–EULAR criteria as reference standard.

Test	Sensitivity	Specificity	PPV	NPV
	% (95% CI)	% (95% CI)	% (95% CI)	% (95% CI)
SGUS ≥2 in ≥1 gland	84.0 (70.9–92.8)	92.0 (80.8–97.8)	91.3 (80.3–96.4)	85.2 (75.2–91.6)
≤0.1 mL/min	92.0 (80.8–97.8)	48.0 (33.7–62.6)	63.9 (57.3–70.0)	85.7 (69.2–94.1)
Schirmer’s test ≤5 mm	65.3 (50.4–78.3)	52.0 (37.4–66.3)	57.1 (48.9–70.9)	60.5 (48.9–70.9)
Anti–SS-A antibody positive	87.8 (75.2–95.3)	100.0 (92.9–100.0)	100.0 (91.8–100.0)	89.3 (79.8–94.6)

CI, confidence interval; PPV, positive predictive value; NPV, negative predictive value; Diagnostic performance was calculated using the 2016 ACR/EULAR classification criteria as the reference standard; Confidence intervals were estimated using the exact (Clopper– Pearson) method; The high specificity observed at higher SGUS cut-off values may be related to the limited sample size.

Abbreviations: SGUS, salivary gland ultrasonography; UWSFR, unstimulated whole salivary flow rate; PPV, positive predictive value; NPV, negative predictive value; CI, confidence interval.

#### ROC analysis of glandular involvement

**Figure 2.**
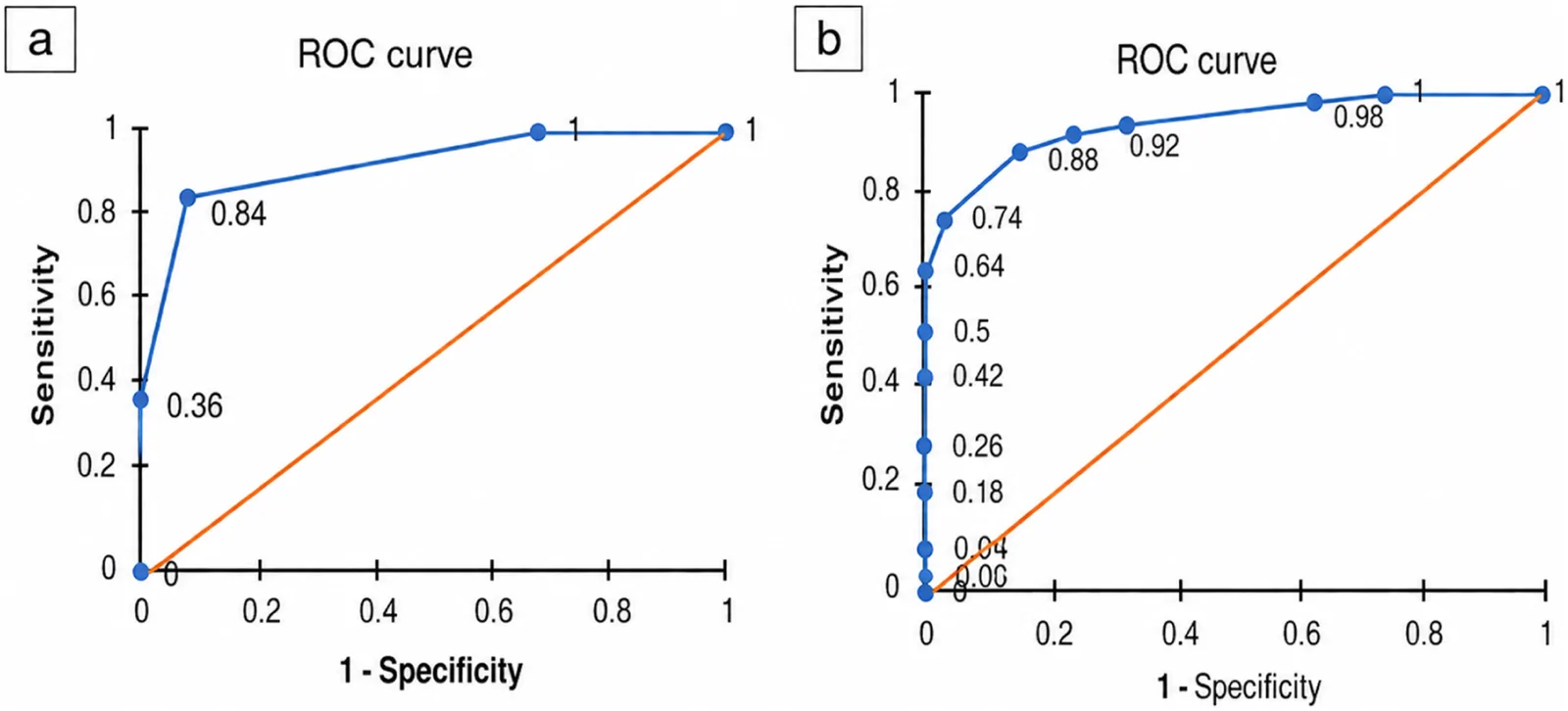
Receiver operating characteristic (ROC) curves of salivary gland ultrasonography (SGUS) for the diagnosis of Sjögren’s disease using the 2016 ACR/EULAR classification criteria as the reference standard. (a) Highest individual gland score (AUC = 0.92; 95% CI, 0.87–0.97; optimal cutoff: grade ≥2; sensitivity: 84%; specificity: 92%). (b) Total SGUS score (AUC = 0.94; 95% CI, 0.90–0.99; optimal cutoff: ≥4; sensitivity: 88%; specificity: 86%).

A ROC curve was generated to evaluate the diagnostic performance of SGUS, with the 2016 ACR/EULAR classification criteria as the reference standard (Figure 2). The highest individual gland score demonstrated excellent diagnostic accuracy, with an area under the curve (AUC) of 0.92 (95% CI, 0.87–0.97). An optimal cutoff of grade ≥2 yielded a sensitivity of 84% and a specificity of 92%. The total SGUS score demonstrated slightly better diagnostic performance, with an AUC of 0.94 (95% CI, 0.90–0.99). An optimal cutoff ≥4 yielded a sensitivity of 88% and a specificity of 86% (Figure 2).

Combined gland analysis also demonstrated excellent diagnostic performance. The combination of the right parotid and right submandibular glands achieved the highest diagnostic accuracy (AUC = 0.95; 95% CI, 0.91–0.99), followed by the combination of all four major salivary glands (**AUC** = 0.94; 95% CI, 0.90–0.99) and the left parotid and left submandibular glands (AUC = 0.93; 95% CI, 0.89–0.99).

## Discussion

This study evaluated the diagnostic performance of SGUS using the OMERACT semi-quantitative scoring system.^7^ It demonstrated good diagnostic performance in differentiating SjD from non-SjD causes of sicca symptoms. These findings are consistent with previous reports supporting the diagnostic value of SGUS in assessing SjD.^7–9^

Significant differences in SGUS scores were observed between the SjD and non-SjD groups, consistent with earlier studies.^7–9,18^ ROC analysis demonstrated high diagnostic performance for both the highest individual gland score and the total gland score, with slightly higher accuracy for the total score, supporting the additional value of multiglandular assessment.^8,18^

Previously proposed thresholds (SGUS ≥2 in at least one gland) were also supported and consistent with earlier studies.^8,19^ However, these thresholds should be interpreted as supportive rather than standalone diagnostic criteria and should be used in combination with clinical and serological findings. Mild (grade 1) changes observed in some non-SjD patients indicate limited specificity of early structural abnormalities and highlight the need for cautious interpretation.^8,18^

No significant differences in SGUS scores were observed between primary and secondary SjD, consistent with previous reports.^8,18^ However, subgroup analyses were limited by sample size, and these findings require validation in larger cohorts.

SGUS provides structural information on the salivary glands and complements functional tests such as Schirmer’s test and UWSFR.^18,19^ In this study, SGUS demonstrated higher specificity than functional tests and showed diagnostic performance numerically similar to that of anti-SSA antibodies. However, no direct statistical comparisons were performed; therefore, these findings should be interpreted descriptively.

The 2016 ACR/EULAR classification criteria remain the reference standard for the classification of Sjögren disease.^3^ Within this framework, SGUS may serve as a valuable adjunct, particularly in seronegative patients or in those with inconclusive clinical and serological findings.^18–23^ Recent evidence from an independent cohort further supports the clinical utility of SGUS as a readily available, non-invasive imaging modality that complements conventional diagnostic investigations and may facilitate earlier recognition of SjD.^23^

Delayed recognition of sicca symptoms was common, consistent with previous observations in SjD. Disease activity was not assessed; therefore, no conclusions can be drawn regarding its relationship with symptom duration. SGUS represents a non-invasive imaging modality that may support earlier diagnostic evaluation in suspected SjD.

### Limitations

This study has several limitations. First, it was conducted at a single center, and the sample size was relatively small for subgroup analyses, which may limit the generalizability of the findings. Second, only 9 patients underwent MSGB, as the procedure was performed only when clinically indicated in routine practice due to its invasive nature. Furthermore, MSGB was performed only in selected anti-SSA–negative patients or those with persistent diagnostic uncertainty rather than systematically in all seronegative patients. Consequently, the limited number of biopsy-confirmed cases restricted a comprehensive comparison between SGUS and minor salivary gland histopathology. It may limit the generalizability of the findings to settings where MSGB is routinely performed in a broader seronegative population. Third, SGUS is an operator-dependent imaging modality and may be subject to interobserver variability, although independent blinded image interpretation and consensus scoring were used to minimize this bias. Fourth, disease duration was self-reported and may therefore be subject to recall bias. Fifth, disease activity and long-term clinical outcomes were not evaluated. Finally, the cross-sectional study design precludes causal inference and assessment of SGUS prognostic value. Although SGUS was excluded from the reference standard classification process to minimize incorporation bias, the use of the 2016 ACR/EULAR classification criteria rather than an expert consensus diagnosis may still have influenced the estimated diagnostic performance. Therefore, larger prospective multicenter longitudinal studies are warranted to validate these findings and further define the role of SGUS in the diagnostic evaluation of Sjögren disease.

## Conclusion

SGUS using the OMERACT scoring system demonstrated high diagnostic performance in differentiating SjD from non-SjD of sicca symptoms. Multiglandular assessment showed higher diagnostic accuracy than single-gland evaluation. SGUS is a non-invasive imaging modality that may complement the current classification criteria, particularly in patients with negative serology or when MSGB is not feasible. However, SGUS should not be used as a standalone diagnostic test and should be interpreted alongside clinical, serological, and other objective findings to minimize the risk of misclassification. Further large-scale prospective multicenter studies are needed to better define its role in future diagnostic and classification frameworks.

## Supplemental material


Supplemental Material - Diagnostic performance of salivary gland ultrasonography using the OMERACT scoring system in Sjögren disease: A cross-sectional diagnostic accuracy study
Supplemental Material for Diagnostic performance of salivary gland ultrasonography using the OMERACT scoring system in Sjögren disease: A cross-sectional diagnostic accuracy study by Md. Ismail Hossain, Minhaj Rahim Choudhury, Muhammad Shoaib Momen Majumder, Md. Nahiduzzamane Shazzad, Mohammad Abul Kalam Azad, Md. Masudul Hassan, Rijwan Bhuiyan and Md. Shohel Hasan in Therapeutic Advances in Musculoskeletal Disease.

## Data Availability

The datasets generated and/or analyzed during the current study are available from the corresponding author on reasonable request.*

## Appendix

### Abbreviations

ACR/EULAR: American College of Rheumatology/European Alliance of Associations for Rheumatology
ANA: Antinuclear antibody
anti-CCP: anti-cyclic citrullinated peptide antibody
AUC: Area under the receiver operating characteristic curve
BMU: Bangladesh Medical University
CLIA: Chemiluminescence immunoassay
CRP: C-reactive protein
dsDNA: double-stranded DNA
ENA: Extractable nuclear antigen
ESR: Erythrocyte sedimentation rate
HEp-2: Human epithelial type 2
MSGB: Minor salivary gland biopsy
NHL: Non-Hodgkin lymphoma
NPV: Negative predictive value
OMERACT: Outcome Measures in Rheumatology
PG: Parotid gland
PPV: Positive predictive value
ROC: Receiver operating characteristic
SGUS: Salivary gland ultrasonography
SjD: Sjögren disease
SMG: Submandibular gland
SSA: Anti-Sjögren syndrome-related antigen A (Ro) antibody
SSB: Anti-Sjögren syndrome-related antigen B (La) antibody
UWSFR: Unstimulated whole salivary flow rate
