# Supplementary material for: Diagnostic performance of salivary gland ultrasonography using the OMERACT scoring system in Sjögren disease: A cross-sectional diagnostic accuracy study
Source: Ther Adv Musculoskelet Dis. 2026 Aug 27;18:1759720X261480003. doi: 10.1177/1759720X261480003 (PMC13525324; doi:10.1177/1759720X261480003)
Supplement: Supplementary file 1 [file sj-pdf-1-tab-10.1177_1759720X261480003.pdf]

Supplementary File 1. STROBE Checklist

Title: *Diagnostic Performance of Salivary Gland Ultrasonography Using the OMERACT Scoring System in Sjögren Disease: A Cross-Sectional Diagnostic Accuracy Study*

Section/Topic	Item	STROBE Recommendation	Reported on Page(s)*
**Title and Abstract**	**1a**	Indicate the study's design in the title or abstract	1
	**1b**	Provide an informative and balanced abstract	1–2
**Introduction**	**2**	Explain the scientific background and rationale	3–4
	**3**	State specific objectives and hypotheses	4
**Methods**	**4**	Present key elements of study design early in the paper	5
	**5**	Describe setting, locations, and relevant dates	5
	**6a**	Describe eligibility criteria and participant selection	5–6
	**6b**	Matching criteria (if applicable)	N/A
	**7**	Clearly define outcomes, exposures, predictors, confounders and diagnostic reference standard	7–10
	**8**	Data sources and measurement methods	7–10
	**9**	Describe efforts to address potential sources of bias	8–10
	**10**	Explain how study size was determined	10
	**11**	Explain handling of quantitative variables	11
	**12a**	Statistical methods	10–11
	**12b**	Methods used to examine subgroups/interactions	11
	**12c**	Handling of missing data	11
	**12d**	Sampling strategy (if applicable)	N/A
	**12e**	Sensitivity analyses	N/A
**Results**	**13a**	Report participant numbers at each stage	12
	**13b**	Give reasons for non-participation/exclusion	12
	**13c**	Consider use of a flow diagram	Figure 1
	**14a**	Describe participant characteristics	12–13 (Table 1)
	**14b**	Indicate missing data	12
	**15**	Report outcome events or summary measures	13–15
	**16a**	Present main results with estimates and precision	13–15 (Tables 3–4)
	**16b**	Report category boundaries if continuous variables are categorized	13–15

Section/Topic	Item	STROBE Recommendation	Reported on Page(s)*
	**16c**	Translate estimates into clinically meaningful measures (if relevant)	13–15
	**17**	Report other analyses (subgroup, ROC, correlations)	14–15
**Discussion**	**18**	Summarize key results	16
	**19**	Discuss study limitations	17
	**20**	Provide interpretation considering objectives and evidence	16–17
	**21**	Discuss generalizability	17
**Other Information**	**22**	Give funding source and role of funders	19
